# In-Hospital Proximal Femoral Fracture Mortality and Anesthesia: Do the First Postoperative 72 h Matter?

**DOI:** 10.3390/jcm14061885

**Published:** 2025-03-11

**Authors:** Raphael Lotan, Chamad Moayad, Mojahed Sakhnini, Nugzar Rijini, Adam Lee Goldstein, Oded Hershkovich

**Affiliations:** 1 Department of Orthopedic Surgery, Wolfson Medical Center, Ha-Lokhamim St 62, Holon 5822012, Israel; dr.lotan@gmail.com (R.L.); mojahed.sakh@gmail.com (M.S.);; 2 Sackler Faculty of Medicine, Tel Aviv University, Tel Aviv 6997801, Israel; 3 Department of Anesthesia, Wolfson Medical Center, Ha-Lokhamim St 62, Holon 5822012, Israel

**Keywords:** mortality, femur, fracture, anesthesia

## Abstract

**Background/Objectives**: Proximal femoral fractures (PFFs) are a common worldwide ailment, causing high morbidity and mortality among the elderly, with one-year mortality estimated at 15–30%. This study aimed to identify factors influencing in-hospital patient survival after proximal femoral fracture surgery. **Methods**: A retrospective cohort study of patients over 65 admitted to an orthopedic surgery department due to a PFF over five years was carried out. Medical records, surgery reports, anesthesia and post-anesthesia care unit sheets, and laboratory archives were reviewed. **Results**: The study group consisted of 48 patients who died during the first postoperative week, while the control group consisted of 69 patients who were discharged for rehabilitation after a week. The study group was older, less active, and had higher rates of comorbidities. Anesthetic factors, such as the type of anesthesia and admixture of drugs, did not have a significant association with mortality. However, a binary logistic regression showed that age (OR = 1.15, *p* < 0.001), intraoperative lactate levels (OR = 5.86, *p* < 0.001), and post-anesthesia care unit (PACU) overnight stays (OR = 17.54, *p* < 0.001) were significantly associated with early mortality. **Conclusions**: The study highlights the challenge of identifying PFF patients at risk of early mortality and the need to better understand the decision-making algorithm to create a reliable scale that can predict mortality and adjust treatment. In our study, solid mortality parameters were age and intraoperative lactate levels. The most significant parameter was PACU overnight stay, which represents the anesthesiologist’s soft-skill-based decision that is challenging to scale and reproduce.

## 1. Introduction

Proximal femoral fractures (PFFs) are a common worldwide ailment, causing high morbidity and mortality among the elderly [1]. One-year mortality within a year following the fracture is estimated at 15–30% [2,3,4]. The percentage of the elderly population in the Western world is rising with the rise in life expectancy, leading to a consistent increase in PFF incidence. According to estimates, the global incidence of hip fractures in 2050 will be multiplied three times, from 1.6 million cases annually to 4.5 million [1]. It has been shown that morbidity and mortality following PFF are related to multiple modifiable factors. Those factors include treatment optimization for underlying diseases, preoperative treatment, time until surgery, type of anesthesia, time until first physical therapy treatment, getting out of bed after surgery, and many other parameters [2,5,6,7,8,9]. At the same time, those patients also have fixed factors that cannot be changed, such as gender, age, type of fracture, osteoporosis and bone mineral density, underlying diseases, and more.

In developed countries, about 95% of femoral neck fractures undergo surgery since conservative treatment involves high mortality and morbidity rates. Evidence of reduced mortality with less than 48 h of early surgery has driven health systems to support early surgery and penalize late surgery financially [2,3,7,10] despite concern over emergent surgery in fragile elderly. Many surgical risk prediction tools have been developed to assess surgical risk and predict mortality. Such tools include the Orthopaedic Trauma Association (OTA) risk calculator [11], the American Society of Anesthesiologists (ASA) score [12], and the National Surgical Quality Improvement Program (NSQIP) risk calculator [13], which uses age, gender, and comorbidities to calculate the 30-day postoperative mortality risk.

Early in-hospital mortality in elderly PFF patients is a significant concern for healthcare providers [14,15,16]. Several factors have been identified contributing to this mortality, including fracture type, surgery, anesthesia, and comorbidities. Studies have shown that patients with displaced or comminuted fractures have a higher mortality rate than those with non-displaced or non-comminuted fractures [17,18]. Additionally, patients who undergo surgery within 24 h of admission have a lower mortality rate than those who do not [19,20].

Comorbidities are also a factor that can influence early in-hospital mortality. Studies have shown that patients with comorbidities such as diabetes, hypertension, and heart disease have a higher mortality rate than those without these conditions [21]. Additionally, patients with multiple comorbidities have a higher mortality rate [3,9,14,15].

Urwin et al., Parker et al., and McLaren et al. showed that patients who received general anesthesia had a higher mortality rate than those who received regional anesthesia [22,23,24]. Rawal et al. found that patients who received a combination of general and regional anesthesia had a higher mortality rate than those who received only regional anesthesia [25]. On the contrary, Lončarić-Katušin et al., White et al., and Parker et al. showed that the type of anesthesia did not correlate with mortality [26,27,28]. Le-Wendling et al. proved that general anesthesia increases mortality risk in the first three months after surgery and even in the first year after the fracture. Others did not find any relationship with the type of anesthesia [29].

Understanding factors affecting 72 h early postoperative proximal femoral fracture mortality in the elderly is scarce [14,15,30]. Most studies deal with in-hospital mortality that combines pre-surgical, surgical, immediate postoperative, and late postoperative mortality.

The purpose of this study was to investigate factors influencing in-hospital survival among patients aged 65 years and older who underwent proximal femoral fracture surgery and were either discharged within a week or experienced in-hospital mortality. The parameters analyzed included patient comorbidities, preoperative hemodynamic status, intraoperative hemodynamics, type of anesthesia administered, intraoperative medications (hypnotics, muscle relaxants, vasopressors, analgesics, and opiates), and duration of stay in the post-anesthesia care unit (PACU).

## 2. Methods

This retrospective cohort study examined patients over 65 admitted to an orthopedic surgery department between 2014 and 2019 due to isolated proximal femoral fracture, subcapital or pertrochanteric, over a five-year period. Medical records, surgery reports, anesthesia and PACU sheets, and laboratory archives were reviewed. The study cohort consisted of patients who died during the first postoperative week and matched one to two with patients who underwent surgery at the beginning of the same year as the study cohort and were discharged for rehabilitation; thus, the matching process was performed randomly based on the date.

Standard descriptive statistics were used to characterize the study population. Continuous variables are expressed as mean ± standard deviation, while categorical variables are presented as frequencies (percentages). Group differences for continuous data were evaluated using Student’s *t*-test or the Mann–Whitney U test, as appropriate based on data distribution, and the Fisher exact test was applied to compare categorical variables. Univariate analyses were conducted to compare preoperative, intraoperative, and postoperative parameters between the early postoperative mortality (FWM) and successful discharge (FWD) groups and between the subgroups of early (within 72 h) and late (72 h to 1 week) mortality. Binary logistic regression analyses were performed to identify independent predictors of early mortality. The predictive performance of the logistic regression models was assessed by generating receiver operating characteristic (ROC) curves and calculating the area under the curve (AUC) with corresponding standard errors and 95% confidence intervals. A *p*-value < 0.05 was considered statistically significant for all tests. All statistical analyses were performed using SPSS (Statistical Package for the Social Sciences) version 25.0 (IBM Corp., Armonk, NY, USA).

## 3. Results

The study cohort included 63 patients who died during the first postoperative week (=FWM—mortality within the first week) out of 2400 patients with PFF. The control group included one to two matching patients who underwent surgery at the beginning of the same year as the study cohort, from 2014 to 2019, and were discharged for rehabilitation (=FWD—successful patient discharge following the first operative week), thus randomly selected by date. Patients lacking complete data were excluded from the study, leaving 48 patients in the study group (76% enrollment) and 69 patients in the control group (54% enrollment). Eventually, the random matching by date was 1:1.4.

The FWM patients were notably older than their FWD counterparts (86.8 ± 7 vs. 74.7 ± 13.7 years, *p* < 0.0001) and included a significantly higher proportion of females (45.8% vs. 26.1%, *p* = 0.03). Mean height also differed significantly (152.6 ± 29.9 cm for FWM vs. 169.9 ± 8.2 cm for FWD, *p* = 0.001), whereas weight (73.4 vs. 73.6 kg, *p* = 0.951) and BMI (25.5 ± 5.2 vs. 27.1 ± 5.8, *p* = 0.324) showed no significant variation between the two groups. Table 1 compares baseline characteristics at admission between the FWM and FWD groups.

Regarding comorbidities, FWM patients had higher rates of hypertension (87.5% vs. 63.8%, *p* = 0.005), ischemic heart disease (39.6% vs. 21.7%, *p* = 0.037), chronic heart congestion (29.2% vs. 4.4%, *p* < 0.0001), and chronic renal failure (41.7% vs. 13%, *p* < 0.0001). Cardiac valvular disorder was also more frequent in FWM (22.9% vs. 5.8%, *p* = 0.006). By contrast, non-insulin-dependent diabetes mellitus (NIDDM), COPD, and asthma rates did not differ significantly between groups, nor did the prevalence of cerebrovascular accidents.

Ambulatory status differed markedly. A significantly lower percentage of FWM patients were fully ambulating (25% vs. 62.3%, *p* < 0.001), whereas a higher proportion relied on walking aids (56.3% vs. 27.5%). Wheelchair dependence (12.5% vs. 8.7%) and bedridden status (6.3% vs. 1.5%) were also more common among FWM, although the relatively small numbers in these categories limit the conclusions that can be drawn.

Regarding the anesthetic approach, general anesthesia was more common in FWM patients (83.3% vs. 72.5%, *p* = 0.006), while regional techniques were observed in 16.7% of FWM versus 27.5% of FWD. With respect to hypnotic use, FWM had lower rates of “no hypnotic” administration (4.2% vs. 14.5%, *p* < 0.001) and showed a higher incidence of etomidate use (37.5% vs. 5.8%). Propofol was administered to 52.1% of FWM and 68.1% of FWD patients, whereas Midazolam was administered to 27.1% and 43.5%, respectively. Table 2 presents anesthesia-related parameters comparing the FWM and FWD groups.

Muscle relaxant patterns similarly differed: no muscle relaxant was used in 18.8% of FWM and 27.5% of FWD patients (*p* = 0.049). Succinylcholine appeared more frequently among FWM patients (10.4% vs. 2.9%), whereas rocuronium was employed more often in FWD patients (63.8% vs. 52.1%). Atracurium was administered to 22.9% of FWM and 8.7% of FWD patients, and vecuronium was used solely for FWM patients (2.1%).

Vasopressor utilization also showed variation, with 75% of FWM patients receiving none versus 91.3% of FWD patients (*p* = 0.02). Ephedrine was administered to 14.6% of FWM and 5.8% of FWD patients, phenylephrine to 12.5% and 2.9%, and noradrenaline to 4.2% and 0%, respectively.

In terms of anesthetic gasses, none were used in 20.8% of FWM and 26.1% of FWD patients (*p* = 0.005). Isoflurane was given to 66.7% and 71%, sevoflurane to 8.3% and 1.4%, and nitrous oxide to 16.7% and 59.4%, respectively.

Opiate administration did not differ significantly (*p* = 0.663). Fentanyl was used in 85.4% of FWM vs. 82.6% of FWD patients, morphine in 14.6% vs. 14.5%, and tramadol in 14.6% vs. 24.6%. Concerning analgesics (*p* = 0.305), none were administered to 62.5% of FWM vs. 62.3% of FWD patients, whereas paracetamol, dipyrone, and diclofenac were used in varying proportions across the two groups.

When analyzing anesthetic-related factors, most FWM and FWD patients underwent general anesthesia; however, the FWM group underwent surgery under general anesthesia more than the FWD group, with rates of 83.3% versus 72.5%, respectively, *p* = 0.006 (Table 2). The admixture of hypnotics, muscle relaxants, vasopressors, and anesthetic gasses differed for FWM and FWD patients, 0.001 < *p* < 0.049 (Table 2), but we found no association between the anesthetic regime and mortality.

Table 3 outlines the patients’ cardiovascular and laboratory parameters measured in the preoperative, intraoperative, and immediate postoperative periods, comparing the FWM and FWD groups. Systolic blood pressure was lower in FWM patients (137.3 ± 22.3 vs. 149.1 ± 0 mmHg, *p* = 0.006), while diastolic blood pressure showed no statistically significant difference (76.6 ± 12 vs. 80.3 ± 0.5 mmHg, *p* = 0.10). Mean arterial pressure (MAP) was also lower in FWM patients (96.8 ± 13.3 vs. 103.3 ± 0 mmHg, *p* = 0.01). Heart rate did not differ significantly (81 ± 15 vs. 80.3 ± 12.5 bpm, *p* = 0.79). Oxygen saturation on room air was lower among FWM patients (91.5 ± 5.6% vs. 94.2 ± 3.3%, *p* = 0.001). Hemoglobin levels were 11.7 ± 1.4 mg% for FWM patients and 12.8 ± 1.7 mg% for FWD patients (*p* < 0.001).

Intraoperative parameters: More FWM patients had an arterial line placed (29.2% vs. 11.6%, *p* = 0.02). The proportion of patients with MAP within 70–100 mmHg was comparable in both groups (83.3% vs. 78.3%, *p* = 0.39). MAP > 100 mmHg was observed in 12.5% of FWM vs. 20.3% of FWD patients, while MAP < 70 mmHg occurred in 4.2% and 1.5%, respectively. Deviation from normal MAP (minutes spent outside the 70–100 mmHg range) did not differ significantly (14.1 ± 18.2 vs. 20.3 ± 0.8, *p* = 0.21). Average heart rates between 60 and 90 bpm occurred in 70.8% of FWM patients and 85.5% of FWD patients (*p* = 0.09). Hemoglobin levels during surgery were lower in FWM patients (9.9 ± 1.5 vs. 11 ± 1.7 mg%, *p* = 0.002), although the pre-intraoperative hemoglobin difference did not differ (1.7 ± 1.3 vs. 1.5 ± 0 mg%, *p* = 0.48). FWM patients received more packed red blood cells than FWD patients (0.6 ± 0.8 vs. 0.1 ± 0.4 units, *p* < 0.001). Serum sodium, potassium, calcium, and lactate levels were generally similar across groups.

Postoperative parameters: Crystalloid administration during surgery and recovery was higher in FWM patients (2056.9 ± 1744.2 vs. 1485.7 ± 679.8 mL, *p* = 0.02). Urine output did not significantly differ (691.2 ± 722.6 vs. 566.6 ± 453.9 mL, *p* = 0.27). Notably, FWM patients spent substantially longer in the post-anesthesia care unit (622.0 ± 588.9 vs. 179.6 ± 148.0 min, *p* < 0.001), with 50% of the FWM group remaining overnight compared to 7.25% of the FWD group (*p* < 0.001). Upon examining patient preoperative hemodynamic parameters, we found a statistically significant 11.8 mmHg difference in preoperative systolic blood pressure between the FWM and FWD groups, 137.3 mmHg compared to 149.1 mmHg, respectively, *p* = 0.006, but with a wide deviation, making it an impractical prognostic factor. Mean arterial pressure showed a similar pattern, a 6.5 mmHg difference in favor of the FWD group (*p* = 0.01). Preoperative room air saturation difference was significant but useless, 91.5 ± 5.6% versus 94.2 ± 3.3%, respectively, *p* = 0.001. We found a difference of 1.1 g/dL preoperative hemoglobin levels, *p* < 0.001 (Table 3). Intraoperatively, FWM patients tended to have lower hemoglobin levels, 9.9 ± 1.5 versus 11 ± 1.7 g/dL, respectively, *p* = 0.002 (Table 3), but the preoperative–intraoperative hemoglobin difference was not different between the cohorts. The FWM cohort had a higher percentage of preoperative arterial-line insertion, 29.2% versus 11.6%, respectively, *p* = 0.02. However, most of the FWM cohort, 70%, did not have an arterial line during surgery, limiting hemodynamic assessment.

Postoperatively, the FWM and FWD cohorts had similar urinary output, 691.2 ± 722.6 versus 566.6 ± 453.9 mL, respectively, *p* = 0.27 (Table 3), but the FWM received a significantly large volume of crystalloids, 2056.9 ± 1744.2 versus 1485.7 ± 679.8, respectively, *p* = 0.02. A statistically significant difference was found in the length of stay at the PACU, 622 ± 588.9 min for the FWM patients and 179.6 ± 148 min for the FWD patients, *p* < 0.001. The same difference was found for overnight stays at the PACU, 50% and 7.25%, respectively, *p* < 0.001 (Table 3).

When further evaluating the FWM cohort for early postoperative mortality (EPM) within 72 h or during 72 h to a week (late first-week mortality, LFWM), twenty-six patients were included in the EPM cohort and twenty-two in the LFWM cohort. Table 4 summarizes patient characteristics between the early postoperative mortality group (EPM), defined as death within 72 h (n = 26), and the late first-week mortality group (LFWM), defined as death after 72 h but within the first week (n = 22). The EPM group showed a slightly higher mean age (88.2 ± 6.5 vs. 85.2 ± 7.3 years, *p* = 0.14), although this difference was not statistically significant. More females were present in the EPM group (61.4% vs. 27.3%, *p* = 0.02). The mean height was 144.6 ± 33.2 cm in the EPM group versus 169.8 ± 7.5 cm in LFWM (*p* = 0.08). Average body weight (74.4 ± 34.3 vs. 72.1 ± 26.1 kg, *p* = 0.80) and BMI (25.6 ± 6 vs. 25.3 ± 2.9, *p* = 0.91) did not differ significantly. Rates of non-insulin-dependent diabetes mellitus (NIDDM) were lower in EPM (30.8% vs. 59.1%, *p* = 0.049). By contrast, there was no significant difference in hypertension (84.6% vs. 90.9%, *p* = 0.51), chronic heart congestion (23.1% vs. 36.4%, *p* = 0.31), or chronic renal failure (38.5% vs. 45.5%, *p* = 0.71). Ischemic heart disease approached significance, with a lower prevalence in EPM (26.9% vs. 54.6%, *p* = 0.051).

Chronic obstructive pulmonary disease (COPD) was observed less often among EPM patients (3.9% vs. 27.3%, *p* = 0.02). Cardiac valvular disorder (30.8% vs. 13.6%, *p* = 0.159), asthma (0% vs. 4.6%, *p* = 0.27), and cerebrovascular attack (26.9% vs. 9.1%, *p* = 0.12) did not show statistically meaningful differences. Regarding mobility, ambulatory status was similar in both groups (26.9% vs. 22.7% fully ambulating, *p* = 0.95), with comparable distributions across walking aids, wheelchair-bound, and bedridden categories.

Using a binary logistic regression, we were able to identify three parameters associated with early mortality: age (odds ratio = 1.153, *p* < 0.001), intraoperative lactate levels (OR = 5.862, *p* < 0.001), and PACU overnight stay (OR = 17.543, *p* < 0.001). Gender was not found to have a statistically significant impact on mortality (*p* = 0.429). All other preoperative, intraoperative, and postoperative parameters mentioned above were not associated with mortality despite statistically significant differences measured between cohorts. Two separate statistical explorations were conducted to assess how well the logistic model predicted the outcome (e.g., mortality). The first analysis included age, intraoperative lactate levels, PACU overnight stay, gender, height, and CHF as predictors, and the second analysis included the first three former parameters. The first analysis produced a receiver operating characteristic (ROC) curve with an area under the curve (AUC) of 0.976. Generally, an AUC of 0.50 indicates a model no better than random guessing, whereas an AUC of 1.00 signifies a perfect classifier. An AUC of 0.976 is considered excellent, demonstrating that the model’s predicted probability discriminates very effectively between individuals who sustained early mortality and those who did not. Accompanying data show a standard error (SE) of 0.011 and a *p*-value < 0.001, indicating that the model’s high AUC is unlikely to have occurred by chance. Moreover, the 95% confidence interval for the AUC ranges from 0.955 to 0.998, remaining well above 0.50 and affirming the model’s robustness in distinguishing outcomes across the sample.

## 4. Discussion

Previous studies have investigated the parameters influencing early PFF mortality in the elderly [6,9,14,15,19]. Some suggested patient pre-fracture morbidity to be significant [9,21], while others investigated the effect of anesthesia on early patient mortality [23,24,25,26,28,29]. A more pressing concern is the relative scarcity of studies devoted to early in-hospital mortality, specifically within the first postoperative days. While it is well known that proximal femoral fracture surgeries carry elevated risks for older adults, few data comprehensively explore how preoperative, intraoperative, and immediate postoperative parameters intersect to shape survival in the first 72 h. Such granular insight is crucial because early mortality may be driven by distinct mechanisms—acute hemodynamic instability, unrecognized tissue hypoperfusion, or residual anesthetic effects—that differ from factors contributing to longer-term morbidity and mortality. By isolating the roles of anesthesia type, intraoperative events, and early postoperative management, researchers and clinicians may better pinpoint strategies to reduce early hospital deaths following hip fracture surgery, ultimately improving both immediate and long-term patient outcomes.

The results of this study suggest that patients who died within the first postoperative week (FWM) following proximal femoral fractures had higher rates of comorbidities, such as hypertension, ischemic heart disease, chronic heart congestion, renal failure, and cardiac valvular disorders, than those who were successfully discharged (FWD) (Table 1). Additionally, the FWM cohort was older and less active than the FWD cohort (Table 1). The FWM had statistically significant lower room air saturation, systolic blood pressure, hemoglobin levels, and mean arterial pressure (Table 3), but not enough to distinguish between FWM and FWD preoperatively. The two cohorts had the same intraoperative hemodynamic characteristics, except for slightly lower hemoglobin levels and a higher percentage of FWM patients that required packed red blood cell transfusion (Table 3). All these mild differences may have led to the higher rate of arterial lines inserted in the FWM cohort and the longer stay times at the PACU, especially overnight stays, *p* < 0.001 (Table 3).

Analyzing the FWM cohort for mortality within 72 h compared to 72 h to a week showed no differences between these subgroups in patient characteristics, anesthesia regime, and postoperative PACU care, except for a paradoxically lower percentage of NIDDM and COPD in early 72 h mortality compared to the later in-hospital mortality.

When examining anesthetic factors, most FWM and FWD patients underwent general anesthesia; however, the percentage of FWM patients who underwent surgery under general anesthesia was higher than the FWD cohort. Additionally, the admixture of hypnotics, muscle relaxants, vasopressors, and anesthetic gasses differed between FWM and FWD patients; however, no association was found between the anesthetic regime and mortality. An inherent limitation of this study is that different anesthetists treated patients with different practices, leading to a significant limitation: these findings are observational, and further studies are needed to establish causality.

Our findings highlight key factors that contribute to very early mortality among older patients undergoing surgery for proximal femoral fractures (PFF). While the prior literature has identified multiple perioperative risk elements—ranging from preoperative comorbidities to anesthesia-related choices—our results underscore three specific parameters that stand out in predicting early postoperative death. First, advanced age (odds ratio [OR] = 1.153) appears to significantly increase the likelihood of 72 h mortality, consistent with the well-established principle that older patients face more challenging physiologic stress and frequently carry heavier comorbidity burdens. Second, elevated intraoperative lactate levels (OR = 5.862) emerged as an important risk indicator, suggesting that tissue hypoperfusion and metabolic compromise during surgery may be pivotal in driving unfavorable outcomes. Third, the requirement of an overnight stay in the post-anesthesia care unit (PACU) (OR = 17.543) further distinguished high-risk individuals. This may reflect prolonged hemodynamic instability or suboptimal early recovery, underscoring the importance of close, high-acuity monitoring in these patients. All the other preoperative, intraoperative, and postoperative parameters assessed were not associated with mortality despite statistically significant differences measured between cohorts. These findings suggest that the PACU team evaluated patient complexity in a fashion that was not directly measured by this study. This finding should lead to a better definition of the soft skill parameters used by the PACU anesthesiologist to create a reproducible, reliable scale.

It seems that older, less active patients with background morbidity (NIDDM, hypertension, IHD, COPD, congestive heart failure, renal failure) and marginally lower preoperative room air saturation, systolic blood pressure, and hemoglobin levels should be declared as patients at risk for close monitoring and management during the postoperative period, particularly in the PACU with overnight stays. This may be crucial when aiming to reduce in-hospital mortality rates in elderly patients with PFF.

From a clinical perspective, these findings have important implications for patient management and prognostication. The data suggest that the earliest postoperative window—particularly the first 72 h—is critical. Patients at higher risk may show more pronounced physiologic stress (e.g., increased lactate), require greater support (e.g., longer PACU stay), and demonstrate less tolerance to even modest hemodynamic fluctuations. Thus, close monitoring and intensified perioperative management during this narrow interval may improve survival, even though anesthesia per se (general vs. regional) does not conclusively drive these early outcomes. Identifying individuals at heightened risk early—based on age, intraoperative lactate, and prolonged PACU stay—could allow for targeted interventions, such as more meticulous intraoperative hemodynamic control, refined blood-product management, and proactive postoperative support. These measures may improve short-term survival prospects in an already vulnerable population. Furthermore, the strong performance of our logistic model, with an area under the curve (AUC) of 0.976, suggests that these variables collectively offer robust predictive power, allowing clinicians to stratify risk effectively.

Despite these compelling results, our study has several limitations. First, it was conducted at a single institution, potentially restricting the generalizability of our findings. Second, although we analyzed a broad range of preoperative, intraoperative, and postoperative parameters, there may be unmeasured confounders—such as nutritional status or frailty indices—that could further clarify risk. Third, the observational design precludes definitive causal inferences, indicating a need for prospective, possibly multicenter investigations to confirm these associations. Another limitation due to the retrospective nature of this study is that different anesthetists treated patients with different practices regarding anesthetic gasses, muscle relaxants, and opiate administration. Finally, sample sizes for certain subgroups (e.g., specific anesthetic regimens) were limited, reducing statistical power in assessing some of the less frequent variables. Although the cohorts are relatively small compared to large population-based studies, they present the opportunity to measure numerous perioperative parameters unavailable in large data-based studies.

## 5. Conclusions

In conclusion, even today, despite numerous studies, improved monitoring tools, and medical care, identifying proximal femoral fracture patients at risk of early mortality remains challenging. Concrete mortality parameters are the patient’s age and intraoperative lactate levels. The most significant associated parameter is the PACU overnight stay, representing an anesthesiologist’s soft-skill-based decision that is hard to scale and reproduce.

Further research is needed to understand the anesthetist’s decision-making algorithm to produce a reliable, reproducible scale that can better predict mortality and adjust treatment accordingly.

## Figures and Tables

**Table 1:** Patient characteristics on admission.

	FWM	FWD	*p*-Value
Age	86.8 ± 7	74.7 ± 13.7	>0.0001
Gender (Females)	45.8%	26.1%	0.03
Height (cm)	152.6 ± 29.9	169.9 ± 8.2	0.001
Weight (kg)	73.4 ± 30.7	73.6 ± 13.6	0.951
BMI (kg/m^2^)	25.5 ± 5.2	27.1 ± 5.8	0.324
NIDDM	43.8%	37.7%	0.171
Hypertension	87.5%	63.8%	0.005
Ischemic Heart Disease	39.6%	21.7%	0.037
Chronic Heart Congestion	29.2%	4.4%	>0.0001
Chronic Renal Failure	41.7%	13%	>0.0001
COPD	14.6%	7.3%	0.198
Asthma	2.1%	5.8%	0.329
Cardiac valvular disorder	22.9%	5.8%	0.006
Cerebrovascular accident	18.8%	18.8%	0.990
Ambulatory status:			
Ambulating	25.0%	62.3%	>0.001
Walking Aids	56.3%	27.5%	
Wheelchair-bound	12.5%	8.7%	
Bedridden	6.3%	1.5%	

BMI—body mass index, NIDDM—non-insulin dependent diabetes mellitus, COPD—chronic obstructive pulmonary disease.

**Table 2:** Anesthesia characteristics.

	FWM	FWD	*p*-Value
General anesthesia	83.3%	72.5%	0.006
Regional anesthesia	16.7%	27.5%	
Hypnotics:			
None	4.2%	14.5%	<0.001
Propofol	52.1%	68.1%	
Etomidate	37.5%	5.8%	
Midazolam	27.1%	43.5%	
Muscle relaxants:			
None	18.8%	27.5%	0.049
Succinylcholine	10.4%	2.9%	
Rokaronium	52.1%	63.8%	
Atracurium	22.9%	8.7%	
Vikoronium	2.1%	0%	
Vasopressors:			
None	75%	91.3%	0.02
Ephedrin	14.6%	5.8%	
Phenylephrine	12.5%	2.9%	
Noradrenaline	4.2%	0%	
Anesthetic gasses:			
None	20.8%	26.1%	0.005
Isoflurane	66.7%	71%	
Sevoflurane	8.3%	1.4%	
Nitrous oxide	16.7%	59.4%	
Opiates:			
None	12.5%	17.4%	0.663
Fentanyl	85.4%	82.6%	
Morphine	14.6%	14.5%	
Tramadol	14.6%	24.6%	
Analgesics:			
None	62.5%	62.3%	0.305
Paracetamol	27.1%	17.4%	
Dypirone	16.7%	26.1%	
Diclofenac	2.1%	0%	

Mortality within the first week = FWM; successful patient discharge following the first operative week = FWD.

**Table 3:** Patient pre-intraoperative characteristics.

	FWM	FWD	*p*-Value
Preoperative:			
Systolic BP (mmHg)	137.3 ± 22.3	149.1 ± 0	0.006
Diastolic BP (mmHg)	76.6 ± 12	80.3 ± 0.5	0.10
MAP (mmHg)	96.8 ± 13.3	103.3 ± 0	0.01
Heart rate (bpm)	81 ± 15	80.3 ± 12.5	0.79
Room air saturation (%O_2_)	91.5 ± 5.6%	94.2 ± 3.3%	0.001
Hemoglobin (mg%)	11.7 ± 1.4	12.8 ± 1.7	<0.001
Intraoperative:			
Arterial line insertion	29.2%	11.6%	0.02
70 mmHg < MAP < 100 mmHg	83.3%	78.3%	0.39
MAP > 100 mmHg	12.5%	20.3%	
70 mmHg < MAP	4.2%	1.5%	
Deviation from normal MAP (min)	14.1 ± 18.2	20.3 ± 0.8	0.21
60 bpm < Average heart rate < 90 bpm	70.8%	85.5%	0.09
Average heart rate > 90 bpm	22.9%	8.7%	
Average heart rate < 60 bpm	6.3%	5.8%	
Tachycardia event > 90 bpm	33.3%	36.2%	0.24
Bradycardia event < 60 bpm	18.8%	10.1%	
Hemoglobin (mg%)	9.9 ± 1.5	11 ± 1.7	0.002
Pre-Intraoperative Hemoglobin difference (mg%)	1.7 ± 1.3	1.5 ± 0	0.48
Intraoperative red blood cell transfusion	0.6 ± 0.8	0.1 ± 0.4	<0.001
Sodium (mmol/L)	136.7 ± 4.6	136 ± 3.1	0.47
Potassium (mmol/L)	4.1 ± 0.5	4.1 ± 0.6	0.85
Calcium (mg/dL)	1 ± 0.1	1 ± 0.1	0.46
Lactate (mg/dL)	2.6 ± 2.4	1.7 ± 1	0.10
Postoperative:			
Crystalloid infusion (surgery and recovery) (cc)	2056.9 ± 1744.2	1485.7 ± 679.8	0.02
Urinary output (surgery and recovery) (cc)	691.2 ± 722.6	566.6 ± 453.9	0.27
Time in PACU (min)	622.0 ± 588.9	179.6 ± 148.0	<0.001
Overnight in PACU	50%	7.25%	<0.001

Post-anesthesia care unit = PACU; mortality within the first week = FWM; successful patient discharge following the first operative week = FWD; mean arterial pressure = MAP; blood pressure = BP.

**Table 4:** Patient characteristics at admission, early mortality (72 h) versus LFWM cohorts.

	EPM (n = 26)	LFWM (n = 22)	*p*-Value
Age (years)	88.2 ± 6.5	85.2 ± 7.3	0.14
Gender (Females)	61.4%	27.3%	0.02
Height (cm)	144.6 ± 33.2	169.8 ± 7.5	0.08
Weight (kg)	74.4 ± 34.3	72.1 ± 26.1	0.80
BMI (kg/m^2^)	25.6 ± 6	25.3 ± 2.9	0.91
NIDDM	30.8%	59.1%	0.049
Hypertension	84.6%	90.9%	0.51
Ischemic Heart Disease	26.9%	54.6%	0.051
Chronic Heart Congestion	23.1%	36.4%	0.31
Chronic Renal Failure	38.5%	45.5%	0.71
COPD	3.9%	27.3%	0.02
Asthma	0%	4.6%	0.27
Cardiac valvular disorder	30.8%	13.6%	0.159
Cerebrovascular attcak	26.9%	9.1%	0.12
Ambulatory status:			
Ambulating	26.9%	22.7%	0.95
Walking Aids	53.9%	59.1%	
Wheelchair bound	11.5%	13.6%	
Bedridden	7.7%	4.6%	

Early postoperative mortality = EPM; late first-week mortality = LFWM; BMI—body mass index, NIDDM—non-insulin dependent diabetes mellitus, COPD—chronic obstructive pulmonary disease.

## Data Availability

As requested to the corresponding author.

## Abbreviations

Proximal femoral fractures = PFF; Post-anesthesia care unit = PACU; Odds-ratio = OR; Mortality within the first week = FWM; successful patient discharge following the first operative week = FWD; Early Postoperative Mortality = EPM; Late first-week mortality = LFWM, BMI—Body Mass Index, NIDDM—non-insulin dependent diabetes mellitus, COPD—Chronic Obstructive Pulmonary Disease; Mean Arterial Pressure = MAP; Blood pressure = BP.

## References

[1] Bäcker H.C., Wu C.H., Maniglio M., Wittekindt S., Hardt S., Perka C. (2021). Epidemiology of proximal femoral fractures. J. Clin. Orthop. Trauma.

[2] Smektala R., Endres H.G., Dasch B., Maier C., Trampisch H.J., Bonnaire F., Pientka L. (2008). The effect of time-to-surgery on outcome in elderly patients with proximal femoral fractures. BMC Musculoskelet. Disord..

[3] Dzupa V., Bartonicek J., Skala-Rosenbaum J., Príkazský V. (2002). Mortality in patients with proximal femoral fractures during the first year after the injury. Acta Chir. Orthop. Traumatol. Cechoslov..

[4] Forster M.C., Calthorpe D. (2000). Mortality following surgery for proximal femoral fractures in centenarians. Injury.

[5] Howard A., Giannoudis P.V. (2012). Proximal femoral fractures: Issues and challenges. Injury.

[6] Maheshwari R., Acharya M., Monda M., Pandey R. (2011). Factors influencing mortality in patients on antiplatelet agents presenting with proximal femoral fractures. J. Orthop. Surg..

[7] Dorotka R., Schoechtner H., Buchinger W. (2003). The influence of immediate surgical treatment of proximal femoral fractures on mortality and quality of life: Operation within six hours of the fracture versus later than six hours. J. Bone Jt. Surg. Br. Vol..

[8] Fox H., Pooler J., Prothero D., Bannister G. (1994). Factors affecting the outcome after proximal femoral fractures. Injury.

[9] Smith T., Pelpola K., Ball M., Ong A., Myint P.K. (2014). Preoperative indicators for mortality following hip fracture surgery: A systematic review and meta-analysis. Age Ageing.

[10] Simunovic N., Devereaux P., Sprague S., Guyatt G.H., Schemitsch E., DeBeer J., Bhandari M. (2010). Effect of early surgery after hip fracture on mortality and complications: Systematic review and meta-analysis. Can. Med. Assoc. J..

[11] Zuckerman J.D., Koval K.J., Aharonoff G.B., Skovron M.L. (2000). A functional recovery score for elderly hip fracture patients: II. Validity and reliability. J. Orthop. Trauma.

[12] Michel J.-P., Klopfenstein C., Hoffmeyer P., Stern R., Grab B. (2002). Hip fracture surgery: Is the preoperative American Society of Anesthesiologists (ASA) score a predictor of functional outcome?. Aging Clin. Exp. Res..

[13] Sathiyakumar V., Greenberg S.E., Molina C.S., Thakore R.V., Obremskey W.T., Sethi M.K. (2015). Hip fractures are risky business: An analysis of the NSQIP data. Injury.

[14] Myers A.H., Robinson E.G., Natta M.L.V., Michelson J.D., Collins K., Baker S.P. (1991). Hip fractures among the elderly: Factors associated with in-hospital mortality. Am. J. Epidemiol..

[15] Groff H., Kheir M.M., George J., Azboy I., Higuera C.A., Parvizi J. (2020). Causes of in-hospital mortality after hip fractures in the elderly. Hip Int..

[16] Ferris H., Brent L., Coughlan T. (2020). Early mobilisation reduces the risk of in-hospital mortality following hip fracture. Eur. Geriatr. Med..

[17] Nyholm A.M., Palm H., Sandholdt H., Troelsen A., Gromov K., DFDB Collaborators (2020). Risk of reoperation within 12 months following osteosynthesis of a displaced femoral neck fracture is linked mainly to initial fracture displacement while risk of death may be linked to bone quality: A cohort study from Danish Fracture Database. Acta Orthop..

[18] Bjørgul K., Reikerås O. (2007). Outcome of undisplaced and moderately displaced femoral neck fractures: A prospective study of 466 patients treated by internal fixation. Acta Orthop..

[19] Moja L., Piatti A., Pecoraro V., Ricci C., Virgili G., Salanti G., Germagnoli L., Liberati A., Banfi G. (2012). Timing matters in hip fracture surgery: Patients operated within 48 hours have better outcomes. A meta-analysis and meta-regression of over 190,000 patients. PLoS ONE.

[20] Orosz G.M., Magaziner J., Hannan E.L., Morrison R.S., Koval K., Gilbert M., McLaughlin M., Halm E.A., Wang J.J., Litke A. (2004). Association of timing of surgery for hip fracture and patient outcomes. JAMA.

[21] Souza R.C.d., Pinheiro R.S., Coeli C.M., Camargo Jr K.R.d. (2008). The Charlson comorbidity index (CCI) for adjustment of hip fracture mortality in the elderly: Analysis of the importance of recording secondary diagnoses. Cad. Saude Publica.

[22] Urwin S., Parker M., Griffiths R. (2000). General versus regional anaesthesia for hip fracture surgery: A meta-analysis of randomized trials. Br. J. Anaesth..

[23] Parker M.J., Handoll H.H., Griffiths R. (2004). Anaesthesia for hip fracture surgery in adults. Cochrane Database Syst. Rev..

[24] McLAREN A.D., Stockwell M.C., Reid V.T. (1978). Anaesthetic techniques for surgical correction of fractured neck of femur. A comparative study of spinal and general anaesthesia in the elderly. Anaesthesia.

[25] Rawal N. (2000). Combined regional and general anaesthesia. Curr. Opin. Anaesthesiol..

[26] Lončarić-Katušin M., Mišković P., Lavrnja-Skolan V., Katušin J., Bakota B., Žunić J. (2017). General versus spinal anaesthesia in proximal femoral fracture surgery–treatment outcomes. Injury.

[27] White S., Griffiths R., Holloway J., Shannon A. (2010). Anaesthesia for proximal femoral fracture in the UK: First report from the NHS Hip Fracture Anaesthesia Network. Anaesthesia.

[28] Parker M.J., Griffiths R. (2015). General versus regional anaesthesia for hip fractures. A pilot randomised controlled trial of 322 patients. Injury.

[29] Le-Wendling L., Bihorac A., Baslanti T.O., Lucas S., Sadasivan K., Wendling A., Heyman H.J., Boezaart A. (2012). Regional Anesthesia as Compared with General Anesthesia for Surgery in Geriatric Patients with Hip Fracture: Does It Decrease Morbidity, Mortality, and Health Care Costs? Results of a Single-Centered Study. Pain Med..

[30] Clague J.E., Craddock E., Andrew G., Horan M.A., Pendleton N. (2002). Predictors of outcome following hip fracture. Admission time predicts length of stay and in-hospital mortality. Injury.

